# Case Report: Sequencing-supported probable osteoarticular *Mycobacterium avium* complex infection in rheumatoid arthritis

**DOI:** 10.3389/fmed.2026.1954797

**Published:** 2026-09-25

**Authors:** Jing Zhang, Lina Zhang, Sheng-Guang Li, Ji Li, Yadan Zou, Ting Long, Ruohan Yu, Yanfeng Zhang

**Affiliations:** Department of Rheumatology and Immunology, Peking University International Hospital, Beijing, China

**Keywords:** hypogammaglobulinemia, metagenomic next-generation sequencing, *Mycobacterium avium* complex, nontuberculous mycobacteria, rheumatoid arthritis, septic arthritis, tenosynovitis

## Abstract

**Background:**

Osteoarticular *Mycobacterium avium* complex (MAC) infection is uncommon and can resemble inflammatory arthritis during immunosuppression.

**Case description:**

A 52-year-old woman with an erosive seronegative rheumatoid arthritis phenotype and overlap autoimmune features developed progressive polyarticular swelling, wrist tenosynovitis, hip effusions, and low-back pain after cumulative immunosuppression. Investigations showed macrocytic anemia, lymphopenia, pan-hypogammaglobulinemia, marked B-cell depletion without anti-CD20 exposure, and cytomegalovirus DNAemia. Pre-treatment mNGS reported MAC-spectrum signals in hip, wrist, and elbow aspirates, including 355 right-hip reads. A separate pathogen-targeted joint-fluid sequencing report, with no anatomical site stated, detected MAC complex (116,330 normalized sequences; assay-estimated 10⁵ copies/mL). Neither result was interpreted as organism burden. No tissue histopathology, dedicated mycobacterial culture, or phenotypic susceptibility testing was performed. The working diagnosis was multisite sequencing-supported probable multifocal osteoarticular MAC infection without culture confirmation. Several interventions began concurrently. Selected follow-up MR images showed persistent destructive wrist disease and residual joint or periarticular fluid, but no standardized imaging or microbiological response endpoint was available.

**Conclusion:**

In atypical arthritis progressing during immunosuppression, infection-focused sampling should precede further escalation. mNGS can provide an etiologic clue, but culture, histopathology, targeted testing, and longitudinal reassessment remain essential.

## Introduction

*Mycobacterium avium* complex (MAC) comprises slowly growing environmental nontuberculous mycobacteria, including *M. avium*, *M. intracellulare*, and *M. chimaera*. Susceptible hosts may develop tenosynovitis, septic arthritis, osteomyelitis, cutaneous disease, or disseminated infection (1). A systematic review of native-joint MAC infection showed that its indolent, non-specific presentation frequently delayed diagnosis and that synovial biopsy was often needed for confirmation (2).

This diagnostic problem is particularly consequential in rheumatoid arthritis (RA): progressive swelling may be interpreted as treatment-resistant synovitis, leading to additional glucocorticoids, biologic disease-modifying antirheumatic drugs (DMARDs), or targeted synthetic DMARDs. We describe a heavily immunosuppressed patient with an erosive seronegative RA phenotype, overlap autoimmune features, and multisite sequencing-supported probable multifocal osteoarticular MAC infection without mycobacterial culture confirmation. A targeted review of culture-confirmed, non-prosthetic MAC-spectrum musculoskeletal infection in established RA identifies practical signals for changing the clinical question from “Which RA drug next?” to “Could this be infection?”

## Case description

### Patient information and prior course

A 52-year-old woman was admitted in October 2025 with progressive multi-joint swelling and pain and new low-back pain. Raynaud-like color changes of both hands began in 2010; antinuclear and anti-U1 ribonucleoprotein antibodies were positive, and undifferentiated connective-tissue disease was considered. Symmetric inflammatory polyarthritis developed in 2018 and involved the proximal interphalangeal, metacarpophalangeal, wrist, elbow, shoulder, knee, and ankle joints. In 2019, rheumatoid factor and anti-cyclic citrullinated peptide antibodies were negative, antinuclear antibody was positive at 1:160, and anti-U1 ribonucleoprotein antibody was strongly positive. Progressive wrist enlargement, deformity, erosions, near fusion, and severe restriction of motion led to a clinical diagnosis of double-seronegative erosive RA. Sicca symptoms, intermittent oral ulcers, Raynaud phenomenon, and anti-U1 ribonucleoprotein positivity suggested overlap autoimmune features. The available record did not document puffy-hand edema distinct from synovitis, sclerodactyly, creatine phosphokinase elevation, objective muscle weakness, or myositis.

In May 2023, treatment consisted of prednisone, methotrexate, and hydroxychloroquine. Tocilizumab was introduced in August 2023 and was withheld after skin infection and breakdown over the elbow. Recurrent arthritis in 2024 was managed with prednisone up to 15 mg/day, repeated tocilizumab, methotrexate, hydroxychloroquine, and iguratimod. Worsening in March 2025 led to tocilizumab, a right-shoulder intra-articular betamethasone injection, a trial of adalimumab, and a left-knee intra-articular injection. Tocilizumab was restarted in June 2025 with injectable methotrexate and prednisone 12.5 mg/day, but swelling continued intermittently and low-back pain developed before admission. Potential portals or exposures recorded in the history were a chronic non-healing foot wound preceding hallux-valgus corrective surgery, elbow skin breakdown/abscess, and intra-articular glucocorticoid injections. Ethnicity, place of origin, long-term residence, occupational or environmental exposures, and nontherapeutic adjuvant or cosmetic injections were not systematically recorded. Major events are summarized in Table 1.

**Table 1:** Timeline of autoimmune disease, immunosuppression, infection clues, treatment, and follow-up.

Time	Event	Clinical relevance
2010	Raynaud-like hand color changes; antinuclear and anti-U1 ribonucleoprotein antibodies positive; undifferentiated connective-tissue disease considered	Autoimmune background before erosive arthritis
2018–2019	Symmetric polyarthritis involving small and large joints; rheumatoid factor and anti-cyclic citrullinated peptide antibodies negative; progressive destructive wrist disease	Retrospectively compatible with the 2010 ACR/EULAR threshold under documented assumptions; overlap features remained possible
May–September 2023	Prednisone, methotrexate, and hydroxychloroquine followed by tocilizumab; tocilizumab was withheld after elbow skin breakdown/abscess	Inflammatory disease and infection risk overlapped
2024–June 2025	Repeated tocilizumab, iguratimod, methotrexate, hydroxychloroquine, prednisone, adalimumab, and shoulder and knee glucocorticoid injections; persistent or progressive swelling	Escalating immunosuppression during persistent symptoms
October 2025	Polyarticular swelling, wrist tenosynovitis, hip effusions, low-back pain, an L2 imaging abnormality of uncertain etiology, profound hypogammaglobulinemia, B-cell depletion, cytomegalovirus DNAemia, pre-treatment mNGS MAC-spectrum signals in hip, wrist, and elbow aspirates (355 right-hip reads), and a separate pathogen-targeted joint-fluid sequencing result (116,330 normalized MAC sequences; anatomical site not stated)	Multisite sequencing-supported probable multifocal osteoarticular MAC infection without culture confirmation; L2 disease not classified as MAC
October–November 2025	Methotrexate, iguratimod, and further biologic therapy stopped; glucocorticoids adjusted; clarithromycin, ethambutol, rifampicin, adjunctive linezolid, IVIG, and ganciclovir started concurrently	Concurrent infection-focused, immune-supportive, antiviral, and anti-inflammatory changes; causal response not assessable
December 2025	Patient reported less generalized pain and hand/knee swelling; local discomfort persisted; moxifloxacin was prescribed for an undocumented reason, but later medication lists were inconsistent	Chronological symptom report without a microbiological endpoint
January–March 2026	Lower-limb paresthesia led to interruption of several agents; glucocorticoid reduction was followed by hand swelling; anemia worsened during linezolid exposure; linezolid was stopped with moxifloxacin prescribed; selected wrist and hip MR images were obtained in March 2026	Selected images showed persistent destructive wrist disease and residual fluid-signal abnormalities; no formal comparative report or standardized imaging score
April–June 2026	Wrist and ankle symptoms persisted; rifampicin rechallenge ended because of gastrointestinal intolerance; final documented regimen was clarithromycin, ethambutol, and moxifloxacin; cystic swellings persisted	No prespecified clinical or microbiological success/failure endpoint

Outpatient medication records were internally inconsistent: moxifloxacin was prescribed for an undocumented reason in December 2025, the January 2026 list again recorded linezolid, and March 2026 documented linezolid discontinuation with moxifloxacin prescription. MAC, *Mycobacterium avium* complex; mNGS, metagenomic next-generation sequencing; RA, rheumatoid arthritis.

### Index presentation and investigations

At admission, physical examination showed swelling and tenderness of multiple small and large joints, marked bilateral wrist deformity, wrist tendon-sheath thickening with hyperemia on Doppler ultrasound, knee effusion, and low-back pain. Imaging demonstrated severe destructive wrist arthropathy with near ankylosis, extensive wrist synovitis and tenosynovitis, bilateral hip synovial thickening and effusions, fluid and edema in periarticular and pelvic muscle planes, and an L2 compression deformity with focal endplate and vertebral destruction. Bone scintigraphy showed multifocal skeletal uptake (Figure 1). The L2 abnormality was not biopsied or classified as probable MAC osteomyelitis or spondylitis; it remained an imaging finding of uncertain etiology.

**Figure 1:**
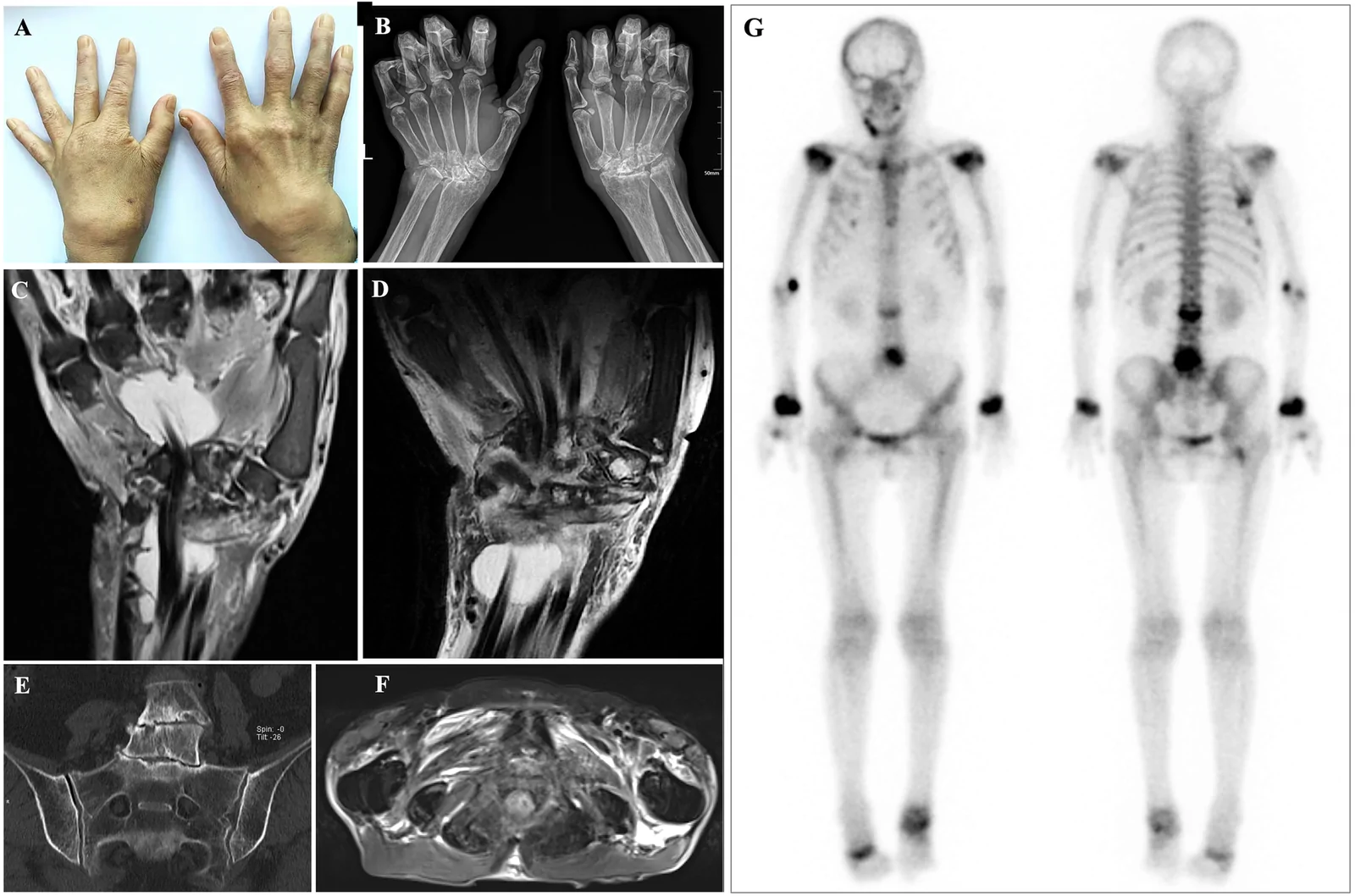
Extensive musculoskeletal involvement in an immunosuppressed patient with an erosive seronegative rheumatoid arthritis phenotype and multisite sequencing-supported probable osteoarticular *mycobacterium avium* complex infection. **(A)** Hands showing swelling of selected proximal interphalangeal and metacarpophalangeal joints and wrists. **(B)** Wrist radiographs showing severe erosive destruction and joint-space narrowing. **(C,D)** Wrist magnetic resonance images showing extensive synovitis, erosive change, and fluid in flexor and extensor tendon sheaths. **(E)** Lumbar imaging showing an L2 compression and destructive abnormality of uncertain etiology; it was not biopsied or classified as MAC osteomyelitis or spondylitis. **(F)** Hip/pelvic magnetic resonance imaging showing hip effusions and edema in periarticular and pelvic muscle planes. **(G)** Bone scintigraphy showing multifocal radiotracer uptake. Imaging is supportive but not pathogen-specific.

At admission, C-reactive protein was 0.3 mg/L. The white-cell count was 5.7 × 10⁹/L, with lymphocytes 0.63 × 10⁹/L. Hemoglobin was 83 g/L with mean corpuscular volume 102.6 fL, and albumin was 30.8 g/L. IgG had been 10.36 g/L in 2019; in 2025, IgG was 3.67 g/L, IgA 0.48 g/L, IgM 0.26 g/L, complement C3 0.62 g/L, CD4-positive T cells 334 cells/microliter, natural-killer cells 22 cells/microliter, and CD19-positive B cells 5 cells/microliter. She had not received anti-CD20 therapy. Plasma cytomegalovirus DNA was 2.40 × 10^3^ copies/mL. Left-knee synovial fluid was clear and pale yellow, with 5,532 leukocytes/microliter, 73.1% polymorphonuclear cells, and 1,000 erythrocytes/microliter. A joint-fluid biochemical specimen from the same laboratory encounter, with no anatomical site stated, showed glucose 3.00 mmol/L, lactate dehydrogenase 1,344 U/L, and adenosine deaminase 17.6 U/L.

Routine bacterial blood cultures and conventional synovial-fluid cultures were negative or non-diagnostic. Before multidrug therapy, hip, wrist, and elbow aspirates underwent metagenomic next-generation sequencing (mNGS); MAC-spectrum signals were reported at all three sites, with 355 MAC reads in the right hip. A separate pathogen-targeted joint-fluid report without an anatomical site label detected MAC complex (116,330 normalized sequences; assay-estimated concentration 10⁵ copies/mL) and lower EBV and cytomegalovirus signals. It described calibration with an internal molecular standard and no covered NTM resistance-gene mutation, but did not provide the platform, specimen processing, negative-control result, positivity threshold, or species assignment beyond MAC complex. Site linkage and assay comparability were not documented; numerical outputs were not combined or interpreted as organism burden. No post-treatment sequencing result, synovial biopsy, histopathology, acid-fast staining, targeted confirmatory polymerase chain reaction, dedicated mycobacterial culture, or phenotypic susceptibility testing was documented. Reasons for the missing confirmatory pathway and residual-specimen status were not recorded (Supplementary Table S1).

### Diagnostic assessment

The differential diagnosis included four non-exclusive explanations: active seronegative RA or overlap connective-tissue disease, probable osteoarticular MAC infection, structural damage from longstanding inflammatory arthritis, and a combination of these processes. Bacterial or fungal infection, tuberculosis, sarcoidosis, IgG4-related disease, lymphoma, and fracture were also considered; biopsy or a dedicated evaluation did not systematically exclude these mimics. The multisite mNGS signals, separate pathogen-targeted joint-fluid result, involved sites, osteoarticular phenotype, and host immune dysfunction supported a working diagnosis of multisite sequencing-supported probable multifocal osteoarticular MAC infection without culture confirmation, but did not establish viable organisms or exclude contamination. Cytomegalovirus was interpreted as reactivation rather than proven viral arthritis because tissue confirmation was unavailable.

### Treatment course and follow-up

Methotrexate, iguratimod, and further biologic therapy were stopped; long-term glucocorticoids were reduced rather than abruptly withdrawn. The pathogen-targeted report recommended clarithromycin 500 mg twice daily, rifampicin 600 mg once daily, ethambutol 1,000 mg once daily, and linezolid 600 mg every 12 h, with adjustment for weight, organ function, and toxicity. The administered regimen used the same agents, but the clinical record listed linezolid 600 mg once daily and weight-based ethambutol and rifampicin. No isolate or phenotypic susceptibility result was available; the negative covered molecular resistance-panel result did not establish phenotypic susceptibility. Comparative drug-selection rationale, maintenance doses, intended duration, and success criteria were not documented. Intravenous immunoglobulin (20 g/day for 3 days) was given as supportive replacement for severe hypogammaglobulinemia, not as MAC-specific therapy; ganciclovir was administered for cytomegalovirus DNAemia.

During the first 2 weeks after these concurrent interventions, pain and joint swelling decreased, although residual pain and morning stiffness persisted; no causal or microbiological response is inferred. A pruritic erythematous rash developed on both lower legs in November and later diminished. By late December, the patient reported less generalized pain and hand and knee swelling, with local discomfort remaining.

Treatment records were internally inconsistent: moxifloxacin was prescribed for an undocumented reason in late December 2025, whereas the January 2026 medication list again included clarithromycin, rifampicin, ethambutol, and linezolid. Lower-limb paresthesia during multidrug exposure prompted interruption of rifampicin and other agents; symptoms lessened, but the dechallenge did not identify a responsible drug. A self-directed glucocorticoid reduction was followed by left-hand swelling, which lessened after the previous dose was restored. In March, anemia worsened while linezolid was being used; clinicians stopped linezolid and prescribed moxifloxacin, but causality was not established against baseline macrocytic anemia. Wrist and ankle symptoms occurred while rifampicin was withheld, and a subsequent rechallenge ended because of gastrointestinal intolerance. Herpes zoster also occurred. Selected bilateral wrist and hip MR images obtained in March 2026, approximately 4–5 months after treatment began, showed less conspicuous periarticular fluid-signal abnormalities on the available images, with persistent severe wrist destruction and residual joint and periarticular fluid (Supplementary Figure S1). No formal comparative radiology report, matched MRI sequence set, or standardized imaging score was available; these selected images were not treated as a pathogen-specific or causal response endpoint. By June 2026, the documented regimen was clarithromycin, ethambutol, and moxifloxacin with continued glucocorticoids; wrist and left-ankle pain and dorsal-wrist and prepatellar cystic swellings persisted. The moxifloxacin dose and complete longitudinal records for ophthalmologic surveillance, ECG/QTc, liver and renal function, electrolytes, blood counts, and dose adjustment were unavailable, as were serial inflammatory markers, immunoglobulins, standardized joint counts, and microbiologic endpoints. Persistent or recurrent symptoms therefore could not be assigned reliably to MAC persistence.

## Discussion and focused literature review

### Literature context

A targeted PubMed search was conducted through 25 July 2026 using combinations of “rheumatoid arthritis,” “*Mycobacterium avium* complex,” “*M. avium*,” “*M. intracellulare*,” “*M. chimaera*,” “nontuberculous mycobacteria,” “septic arthritis,” “tenosynovitis,” “dactylitis,” “osteomyelitis,” and “prosthetic joint infection.” The comparison emphasized culture-confirmed MAC-spectrum musculoskeletal infection in established RA and used RA-mimicking, prosthetic-joint, non-RA native-joint, and mNGS-based reports as contextual comparators. The search was purposive and narrative, not a registered systematic review; therefore, no PRISMA flow claims or pooled estimates were made.

The search identified at least eight culture-confirmed, non-prosthetic RA-associated reports (3–10). They encompassed injection-associated shoulder infection, disseminated extremity abscesses with calcaneal osteomyelitis, dactylitis, metacarpophalangeal-joint disease, and hand, wrist, or forearm tenosynovitis under conventional or biologic immunosuppression. Across reports, mycobacterial culture established the diagnosis and tissue pathology frequently showed granulomatous inflammation.

The wider diagnostic spectrum includes MAC infection initially misdiagnosed as seronegative RA (11), culture-confirmed RA-associated hip prosthetic-joint infection (12), and a five-case MAC prosthetic-joint series in which three patients had RA (13). Non-RA reports further illustrate indolent hand/wrist tenosynovitis, native-wrist and native-knee infection, an mNGS-supported spinal presentation without culture confirmation, and rice-body tenosynovitis (14–18); a broader review of nontuberculous mycobacterial prosthetic-joint infection adds surgical context (19). Table 2 integrates the RA-associated reports most relevant to the current case. The current case differs because the musculoskeletal distribution was multifocal and the etiologic clue came from multisite mNGS without mycobacterial culture or histopathologic confirmation.

**Table 2:** Comparison of selected rheumatoid arthritis-associated *mycobacterium avium* complex-spectrum bone, joint, and tenosynovial infections with the current case.

Report	Host context	Site and organism	Diagnostic evidence	Management, outcome, and comparison
Murdoch and McDonald, 2007 (3)	Seronegative RA treated with prednisone and azathioprine; bilateral intra-articular glucocorticoid injections	Bilateral injection-site cellulitis and left native-shoulder septic arthritis; *M. avium-intracellulare*	Skin biopsy showed granulomas; skin and joint cultures were positive	Debridement and prolonged multidrug therapy; relapse after treatment interruption and permanent joint damage
Yaita et al., 2017 (4)	RA treated with long-term prednisolone and bucillamine	Disseminated extremity abscesses and calcaneal osteomyelitis; *M. intracellulare*	Acid-fast testing, targeted testing, and cultures of pus or bone were positive	Drainage plus clarithromycin, rifampicin, and ethambutol; fatal outcome
Saraya et al., 2018 (5)	RA treated with prednisolone, methotrexate, and sulfasalazine; worsened after infliximab	Hand/wrist granulomatous tenosynovitis with rice bodies; *M. intracellulare*	Discharge and tissue culture, targeted polymerase chain reaction, and histopathology	Synovectomy/debridement and multidrug therapy; recurrence years later, followed by response to retreatment
Watanabe et al., 2020 (6)	Seronegative RA treated with methotrexate, tacrolimus, and prednisolone	Finger, thumb, and wrist tenosynovitis; *M. chimaera*	Granulomatous histopathology, two tissue cultures, and mass spectrometry	Extensive debridement plus rifampicin, ethambutol, and clarithromycin; still receiving therapy at 4 months
Matcuk et al., 2020 (7)	RA treated with methotrexate, prednisone, and adalimumab	Hand/wrist horseshoe abscess with rice bodies; *M. avium-intracellulare*	Operative cultures were positive	Irrigation, debridement, and synovectomy; emphasizes MRI-defined extent and operative culture
Shimizu et al., 2022 (8)	RA treated with methotrexate	Middle-finger dactylitis and flexor tenosynovitis; *M. intracellulare*	Biopsy tissue culture later became positive	Rifampicin, ethambutol, and clarithromycin; marked improvement by 6 months
Hanaoka, 2025 (9)	Well-controlled RA treated with methotrexate; local glucocorticoid injection and gardening exposure	Index-finger metacarpophalangeal joint, flexor tendon, and bone marrow; *M. intracellulare*	Synovectomy culture, susceptibility testing, and granulomatous histopathology	Synovectomy and prolonged triple therapy; no recurrence but residual motion limitation
Hagiya et al., 2025 (10)	RA treated sequentially with conventional and biologic therapies	Forearm/hand flexor tenosynovitis with progressive marrow and soft-tissue involvement; *M. intracellulare*	Culture of resected tissue was positive	Repeat tenosynovectomy and prolonged multidrug therapy complicated by toxicities; improvement with residual dysfunction
Ingraham et al., 2017 (12)	Severe RA treated with prednisone, leflunomide, abatacept, and prior infliximab	Hip prosthetic-joint infection; *M. avium-intracellulare*	Acid-fast smear and cultures of aspirated collections were positive	Limited irrigation/debridement and antimycobacterial therapy; clinical deterioration and death after hospice transition
Dobos et al., 2022 (13)	Five MAC prosthetic-joint infections; three patients had RA	Prosthetic hip or knee infection; MAC	Repeated positive cultures in most cases	Prolonged multidrug therapy and surgery; demonstrates the difficult medical-surgical course of prosthetic disease
Current case	Erosive seronegative RA phenotype/overlap features; multiple conventional and biologic therapies; profound immune dysfunction	Probable multifocal native-joint and tendon-sheath disease; L2 imaging abnormality of uncertain etiology; MAC-spectrum signal	Pre-treatment multisite mNGS signals in hip, wrist, and elbow aspirates, including 355 right-hip reads, plus a separate pathogen-targeted joint-fluid result with 116,330 normalized MAC sequences (site not stated); routine bacterial cultures non-diagnostic; no dedicated mycobacterial culture or tissue confirmation	Concurrent immunosuppression reduction, empirical multidrug therapy, IVIG, and ganciclovir; selected interval MRI without a standardized response endpoint; symptom changes, recurrent symptoms, paresthesia, worsening pre-existing anemia, and gastrointestinal intolerance over 7 months; efficacy and microbiological response not determined

DMARD, disease-modifying antirheumatic drug; MAC, *Mycobacterium avium* complex; mNGS, metagenomic next-generation sequencing; RA, rheumatoid arthritis.

Three recurring patterns emerge. First, local barrier disruption matters: intra-articular injection, previous surgery, environmental exposure, or skin breakdown preceded several infections. Second, tendon-sheath disease was disproportionately represented. Chronic hand or wrist tenosynovitis, rice bodies, draining tracts, or recurrent swelling after an apparent RA-directed response should therefore prompt tissue sampling rather than repeated local glucocorticoid injection. Third, diagnostic delay had consequences ranging from irreversible loss of motion to disseminated or fatal disease. These reports do not establish comparative risk among antirheumatic drugs, but they consistently show that escalating immunosuppression before obtaining specialized microbiology can amplify an unrecognized infection.

### The diagnostic pivot: infection before further escalation

Several red flags argued against automatically labeling this presentation an RA flare: progression despite sequential drug classes, extensive tenosynovitis and effusions, skin breakdown and abscess, repeated intra-articular glucocorticoid exposure, new axial pain, profound hypogammaglobulinemia and lymphocyte-subset abnormalities, and cytomegalovirus reactivation. In this setting, infection-focused sampling should precede another biologic switch.

The case also shows why phenotype alone is insufficient. Tenosynovitis, erosions, effusions, marrow or soft-tissue edema, and radionuclide uptake can occur in both inflammatory and infectious disease. When nontuberculous mycobacteria are suspected, specimens should be explicitly submitted for acid-fast staining, dedicated mycobacterial culture with prolonged incubation, species identification, susceptibility testing, and histopathology. Tissue may be more informative than low-biomass fluid.

The pre-existing rheumatologic diagnosis requires nuance. The 2010 ACR/EULAR RA criteria require clinical synovitis not better explained by another disease and a score of at least 6/10 (20). Retrospectively, the recorded involvement of more than 10 joints including small joints (5 points) and symptoms lasting more than 6 weeks (1 point), with negative rheumatoid factor and anti-cyclic citrullinated peptide antibodies, was compatible with the threshold; contemporaneous formal scoring and the no-better-explanation condition could not be reconstructed. Raynaud phenomenon, anti-U1 ribonucleoprotein antibodies, and polyarthritis prompted consideration of mixed connective-tissue disease, but the available record did not establish puffy-hand edema distinct from synovitis, sclerodactyly, myositis, creatine phosphokinase elevation, or the multi-domain findings needed to confirm Alarcón-Segovia or Kasukawa criteria (21). The safest interpretation is an erosive seronegative RA phenotype with overlap autoimmune features, while recognizing that infection, inflammation, and structural damage may have coexisted.

### What sequencing established—and what it did not

Sequencing supplied an etiologic clue after routine bacterial cultures were unrevealing. Detection in aspirates from several involved sites, the separate high-signal pathogen-targeted joint-fluid result, a compatible osteoarticular phenotype, severe host immune dysfunction, and progression during immunosuppression increased clinical concern. However, the targeted report did not identify its anatomical source, and site labels did not establish the same species, strain, or independent sequence profile across specimens. Detection of microbial DNA does not distinguish viable organisms from nonviable DNA or contamination, and environmental or reagent contamination is a recognized challenge in clinical mNGS (22). An osteoarticular-infection cohort showed that mNGS can add detections to conventional testing, but this does not validate an individual MAC signal (23). Without biopsy, acid-fast stain, targeted confirmatory polymerase chain reaction, dedicated mycobacterial culture, or phenotypic susceptibility testing, the current case remains sequencing-supported probable infection without culture confirmation and should not be shortened to “MAC arthritis.”

Sequencing counts and concentration estimates are assay- and background-dependent and cannot be interpreted as organism burden across different assays without validated controls and a reporting framework. The available record lacked the controls, limit of detection, site linkage, and cross-specimen concordance needed for that interpretation. A negative result in a limited molecular resistance panel does not replace isolate-based phenotypic susceptibility testing. When clinically feasible, a positive signal should therefore trigger repeat aspiration or synovial/tenosynovial biopsy for acid-fast staining, prolonged mycobacterial culture, species identification, targeted molecular confirmation, and susceptibility testing.

### Host immune dysfunction

The immune phenotype was notable: pan-hypogammaglobulinemia, near-absence of circulating B cells without anti-CD20 exposure, CD4 lymphopenia, reduced natural-killer cells, and opportunistic viral events. IgG had been within the laboratory range in 2019, favoring an acquired decline, but primary antibody deficiency could not be excluded. Common variable immunodeficiency could not be diagnosed because repeated pre-IVIG immunoglobulins, functional vaccine responses, and systematic exclusion of secondary causes were unavailable; isolated IgG subclass deficiency would not explain the markedly low total IgG (24). Medication effects, renal or gastrointestinal protein loss, nutritional factors, marrow or lymphoproliferative disease, and other acquired disorders remained possible. HIV testing was not documented in the available record. Long-term reassessment should include serial immunoglobulins and lymphocyte subsets after stabilization, medication review, evaluation for protein loss, hematologic testing when indicated, HIV testing if not already completed, and immunology-directed functional antibody assessment recognizing that prior IVIG complicates interpretation.

### Treatment reasoning and uncertainty

Evidence for extrapulmonary osteoarticular MAC treatment derives mainly from case reports and small series; pulmonary guidance is not an osteoarticular-specific standard (25, 26). That guidance generally favors azithromycin for tolerance and fewer interactions while accepting clarithromycin as an alternative (26). In this case, the pathogen-targeted report recommended clarithromycin, rifampicin, ethambutol, and linezolid, but did not explain the choice of clarithromycin over azithromycin or the specific reason for adding linezolid. The report's negative molecular resistance-panel result did not establish phenotypic susceptibility. Moxifloxacin was prescribed in December for an undocumented reason and again in March when linezolid was stopped during evaluation of worsening anemia; it should not be viewed as an evidence-equivalent replacement. Without isolate-based susceptibility testing, a prespecified duration, complete monitoring records, or microbiological endpoints, regimen adequacy and treatment success or failure could not be determined. Concurrent ganciclovir, IVIG, glucocorticoid adjustment, and withdrawal of other immunosuppressants preclude attribution of symptom changes to one intervention.

The follow-up course illustrates the uncertainty of both toxicity and disease attribution. Paresthesia developed during exposure to several agents; linezolid- or ethambutol-associated neurotoxicity, other drugs, lumbar disease, and non-drug causes remained possible. The temporal change after interrupting several agents did not isolate rifampicin. Anemia preceded antimycobacterial therapy and later worsened during linezolid exposure; stopping linezolid reflected clinical suspicion rather than proof of myelosuppression. Recurrent wrist and ankle symptoms could represent active inflammatory disease, structural damage, probable infection, or a combination. Glucocorticoid-associated fluctuation supported persistent inflammatory activity, but no clinical symptom pattern established MAC persistence. Repeat high-yield sampling and imaging would be preferable to empirical escalation. Decisions about restarting RA therapy require reassessment of infection control, toxicity, and the immune defect.

Source control deserves explicit consideration because published osteoarticular and tendon-sheath cases often required biopsy, synovectomy, drainage, or debridement (1–19). In the current episode, only percutaneous aspirations were documented; no formal orthopedic consultation, biopsy, synovectomy, operative drainage, or debridement was recorded, and whether these options were considered could not be established. Multidisciplinary reassessment is warranted if a focal effusion, tendon sheath, abscess, or necrotic focus persists.

### Strengths, limitations, and take-away lessons

The report's strengths are multisite sampling, explicit immune phenotyping, a detailed 7-month chronology, selected interval MR images, and conservative diagnostic classification. Its principal limitation is the absence of culture, histopathology, targeted confirmatory testing, phenotypic susceptibility testing, and complete sequencing metadata. Reasons for the missing confirmatory pathway and residual-specimen status were not documented. The rheumatologic and immune diagnoses also remained incompletely resolved, and sarcoidosis, IgG4-related disease, lymphoma, protein loss, and hematologic causes were not systematically excluded. Follow-up lacked standardized joint counts, serial laboratory data, a formal comparative radiology report or standardized imaging score, and microbiological endpoints. Consequently, microbiologic cure, optimal treatment duration, treatment efficacy, toxicity attribution, and the safety of reintroducing antirheumatic therapy remain undetermined. The literature review was focused rather than exhaustive.

For practice, atypical arthritis progressing during immunosuppression should trigger an infection-focused reassessment before further escalation. mNGS can accelerate that pivot, but should initiate rather than replace confirmatory tissue-based investigation. During follow-up, infection, inflammatory activity, structural disease, drug exposure, and toxicity should be reassessed as non-exclusive explanations (Figure 2).

**Figure 2:**
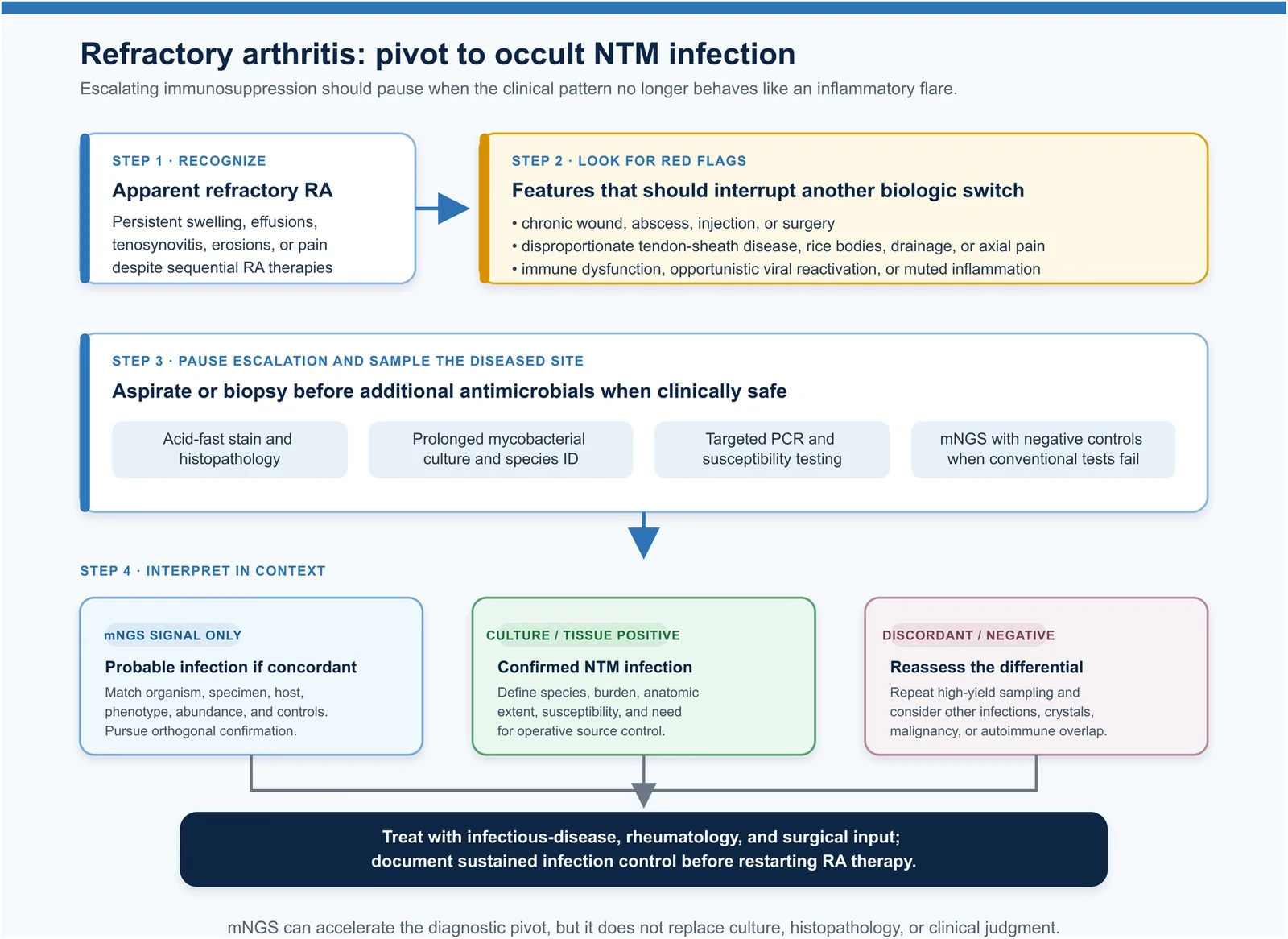
Diagnostic pivot when apparently refractory rheumatoid arthritis under immunosuppression may conceal nontuberculous mycobacterial infection. The algorithm emphasizes red flags, infection-focused tissue sampling, cautious interpretation of metagenomic next-generation sequencing, multidisciplinary antimycobacterial management, and reassessment before restarting rheumatoid arthritis therapy.

## Patient perspective

During follow-up, the patient reported less generalized pain and hand and knee swelling by late December 2025, although local discomfort remained. She subsequently described lower-limb numbness and recurrent wrist and ankle pain during treatment changes. A glucocorticoid reduction was followed by renewed left-hand swelling, which lessened after the previous dose was restored. By June 2026, she remained on antimicrobial therapy with wrist and left-ankle discomfort. These observations describe chronology and do not establish the effect of any single intervention.

## Data Availability

The raw data supporting the conclusions of this article will be made available by the authors, without undue reservation.

## Supplementary material

Supplementary Figure S1 Selected interval magnetic resonance images. **(A,B)** Right wrist at baseline and approximately 4.5 months after treatment initiation. **(C,D)** Left wrist at baseline and approximately 4.5 months after treatment initiation. **(E,F)** Hips at baseline and approximately 4.5 months after treatment initiation. On the available images, periarticular fluid-signal abnormalities were less conspicuous at follow-up, while severe destructive wrist abnormalities and residual joint or periarticular fluid persisted. The displayed images were selected from different acquisitions and were not accompanied by a formal comparative radiology report or standardized score; they are not a pathogen-specific or causal treatment endpoint.
