# Supplementary material for: Case Report: Sequencing-supported probable osteoarticular Mycobacterium avium complex infection in rheumatoid arthritis
Source: Front Med (Lausanne). 2026 Sep 25;13:1954797. doi: 10.3389/fmed.2026.1954797 (PMC13649503; doi:10.3389/fmed.2026.1954797)
Supplement: Supplementary file 1 [file Table1.docx]

**Supplementary Table S1. Available mNGS and pathogen-targeted sequencing information**

*Manuscript 1954797 — sequencing-supported probable osteoarticular Mycobacterium avium complex infection*

**Scope:** This table reports only information available in the de-identified clinical summary, source presentation, and pathogen-targeted laboratory report. Unavailable assay metadata are labeled as not documented rather than reconstructed.

| **Assay and specimen/site** | **Timing relative to multidrug therapy** | **Reported finding** | **Quantitative output** | **Species/site concordance** | **Available assay details** | **Orthogonal/phenotypic confirmation** | **Source limitation** |
| --- | --- | --- | --- | --- | --- | --- | --- |
| mNGS; right-hip aspirate | Before therapy | MAC-spectrum signal; CMV DNA also reported | 355 MAC reads | Not documented | Platform, processing, library method, negative-control result, positivity threshold, and environmental/reagent controls not documented | No dedicated mycobacterial culture, AFB stain, targeted confirmatory PCR, histopathology, or phenotypic DST documented | Primary laboratory report unavailable; CMV was listed as 1,579 reads in the source presentation |
| mNGS; wrist aspirate | Before therapy | MAC-spectrum signal; CMV DNA also reported | Not documented | Not documented | Platform, processing, library method, negative-control result, positivity threshold, and environmental/reagent controls not documented | No dedicated mycobacterial culture, AFB stain, targeted confirmatory PCR, histopathology, or phenotypic DST documented | Primary laboratory report unavailable |
| mNGS; elbow aspirate | Before therapy | MAC-spectrum signal reported; CMV status not documented | Not documented | Not documented | Platform, processing, library method, negative-control result, positivity threshold, and environmental/reagent controls not documented | No dedicated mycobacterial culture, AFB stain, targeted confirmatory PCR, histopathology, or phenotypic DST documented | Specimen-level primary report unavailable |
| Pathogen-targeted sequencing; joint fluid, anatomical site not stated | Before therapy; sampled in October 2025 | MAC complex; EBV and CMV also detected; no covered NTM resistance-gene mutation reported | MAC 116,330 normalized sequences, estimated 10⁵ copies/mL; EBV 860 and CMV 291 normalized sequences, each estimated <10³ copies/mL | Species assignment remained at MAC-complex level; linkage to the three site-labeled mNGS specimens not documented | Concentration estimate used a known molecular internal control and amplification calibration; platform, pre-analytic processing, negative-control result, and positivity threshold not reported; assay scope described as 3 NTM drugs/4 genes | Molecular panel result only; no culture-based or phenotypic DST, histopathology, AFB stain, or independent targeted PCR documented | Report cautioned that the molecular result was not the sole standard for resistance diagnosis; numerical output is not combined with mNGS read counts |
| Post-treatment sequencing assessment | Not documented | No post-treatment mNGS or pathogen-targeted sequencing result documented | Not applicable | Not applicable | Not applicable | No post-treatment microbiological endpoint documented | Whether a post-treatment specimen was submitted was not documented |

**Notes.** All available sequencing results preceded multidrug therapy. The 355 mNGS reads, normalized sequence counts, and assay-estimated concentration came from different reporting systems and are not combined or interpreted as organism burden. The record did not establish whether specimens were processed in the same run, whether the targeted joint-fluid specimen came from one of the three site-labeled joints, whether the same species or strain was present across sites, or whether environmental/reagent contamination controls were acceptable. The reason dedicated mycobacterial specimens were not obtained and whether residual stored material remained could not be established. AFB, acid-fast bacillus; CMV, cytomegalovirus; DST, drug-susceptibility testing; EBV, Epstein–Barr virus; MAC, Mycobacterium avium complex; mNGS, metagenomic next-generation sequencing; NTM, nontuberculous mycobacteria; PCR, polymerase chain reaction.
